# Effect of dietary energy levels on productivity, fat deposition, and biochemical parameters of Woorimatdag1 breeder pullets

**DOI:** 10.5713/ab.24.0369

**Published:** 2024-10-28

**Authors:** Hyojun Choo, Chunik Lim, Hyeonkwon Kim, Kangnyeong Heo, Euichul Hong

**Affiliations:** 1 Poultry Research Institute, National Institute of Animal Science, RDA, Pyeongchang 25342, Korea

**Keywords:** Biochemical Parameters, Fat Deposition, Metabolizable Energy, Productivity, Woorimatdag Breeders

## Abstract

**Objective:**

This study aimed to investigate effects of apparent metabolizable energy (AME_n_) levels in diets on productivity, fat deposition, and biochemical parameters of Woorimatdag1 (WMD1) breeder pullets.

**Methods:**

A total of 240 four-week-old WMD1 breeder pullets were divided into four dietary groups with five replicates (12 birds per replicate). These groups had the following dietary energy levels: standard metabolizable energy (SME), SME-200, SME-100, and SME+100 (diets containing 2,800, 2,600, 2,700, and 2,900 kcal AME_n_/kg, respectively). These pullets were provided with diets and water *ad libitum* until 16 weeks old.

**Results:**

Weight gain was significantly (p<0.05) higher in SME-100, SME, and SME+100 groups than in the SME-200 group. SME+100 and SME groups exhibited significantly (p<0.05) improved feed conversion ratio compared to the SME-200 group. Laying ages of 30% egg production occurred significantly (p<0.05) earlier in SME-100, SME, and SME+100 groups than in the SME-200 group. SME and SME+100 groups had significantly (p<0.05) higher liver fat (%) than the SME-200 group. Additionally, the SME+100 group had higher (p<0.05) abdominal fat (%) than other groups. However, blood parameters were not significantly different among dietary groups.

**Conclusion:**

SME-100 (2,700 kcal AME_n_/kg) might be suitable for improving productivity and fat deposition of WMD1 breeder pullets.

## INTRODUCTION

The Global Living Planet Index declined by 68% from 1900 to 2016, primarily due to overfishing, habitat degradation, and loss [1]. This decline, alongside the growth of industries dependent on biological resources, such as food and pharmaceuticals, has heightened awareness of the need for biodiversity conservation and the importance of preserving native species [2,3].

Native chickens possess a remarkable capacity for adaptation, enabling them to thrive and reproduce in traditional environmental conditions [4]. These birds are deeply intertwined with sociocultural communities. Their role in producing high-quality animal proteins underscores their importance in meeting Korea’s growing demand for poultry meat [5]. However, the dominance of foreign companies, which control about 95% of chicken breeds in Korea’s poultry market, poses significant challenges [6]. Such dominance can lead to instability in supply and demand with potential extinction of native genetic resources within the industry [7]. From 1992 to 2006, the National Institute of Animal Science in Korea established pure breeds of Korean Native Chickens (KNCs) [8]. Woorimatdag1 (WMD1), a crossbreed of Black Cornish and Brown KNC male with Rhode Island Red female, is considered a high-quality protein source with a rich flavor [8,9]. However, due to their high production costs, the distribution of WMD1 breeders is very low, accounting for less than 5% of the entire poultry industry in Korea [6]. Accordingly, to enhance economic feasibility, reducing production costs of WMD1 might serve as a strategy to expand the foundation of the KNC industry, which is considered a valuable approach for conserving KNCs [10,11].

Establishing energy requirement in diet for breeder hens is a critical economic imperative within farm operations [12]. Moreover, proficient management of hens necessitates meticulous control of body weight (BW) across growth and reproduction phases [13]. Strategically regulating energy intake throughout each phase holds promise in not only reducing feed costs, but also amplifying the efficiency of chick production [14]. It is imperative to underscore the indispensability of adequate energy intake for breeder hens, as excessive consumption precipitates deposition of adipose tissue in diverse anatomical substrates, including the liver, muscles, and blood vessels [13,15]. Fat deposition detrimentally impacts vital metrics such as production, fertility, hatchability, and feed efficiency [14,16].

Apparent metabolizable energy (AME_n_) has been applied as a standard model for energy requirements in poultry [17,18]. It is regarded as an essential nutrient for practical biological activities and maintenance processes in poultry [19]. However, few research studies have determined optimal levels of AME_n_ to improve both production and quality in the KNC industry [20,21]. Furthermore, AME_n_ levels in dietary formulations for WMD1 breeder hens often rely on nutritional requirements of foreign broiler breeder hens, highlighting the importance of dietary research for WMD1 [6]. In particular, it is known that optimal weight management accompanied by optimal AME_n_ is important for sexual maturity of pullets, aiming to enhance egg production and economic efficiency during the layer phase [22,23].

Therefore, the purpose of the present study was to determine the optimal AME_n_ level in the diet for productive performance, fat deposition, and blood biochemical composition of WMD1 breeder pullets aged 4 to 16 weeks.

## MATERIALS AND METHODS

### Birds and experimental diets

**Figure 1:**
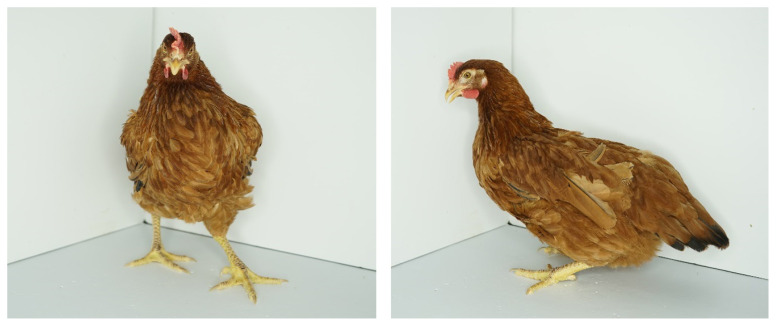
Woorimatdag1 breeder hens.

Experimental protocols were approved by the Institutional Animal Care and Use Committee of the National Institute of Animal Science, Rural Development Administration, Korea (NIAS 2021-0519). In this study, WMD1 breeder hens (Figure 1), a crossbreed between Red-Brown KNC males and Korean Rhode Island Red females, were raised from 4 to 16 weeks of age. A total of 240 four-week-old WMD1 breeder pullets with an average weight of 215±5 g were assigned into 20 floor cages (12 birds per cage, 120 cm×60 cm×40 cm; 600 cm^2^ per bird) under environmentally controlled room conditions. Experimental diets were formulated for broiler breeder pullets through starter phase (4 to 6 weeks of age) and grower phase (7 to 16 weeks of age). Dietary groups consisted of four levels: standard metabolizable energy (SME), SME-200, SME-100, and SME+100) at all phases. The SME diet was formulated to meet nutrient requirements (2,800 kcal AME_n_/kg) of broiler breeder pullets as described in the Korean Feeding Standard for Poultry in the National Institute of Animal Science [18]. SME-200, SME-100, and SME+100 diets were formulated with 2,600, 2,700, and 2,900 kcal AME_n_/kg, respectively. The other nutrient contents, including crude protein (CP), calcium (Ca), and available phosphorus (AP), were formulated to be consistent across all treatments as recommended by Korean Feeding Standard for Poultry in the National Institute of Animal Science [18]. Specifically, diets for 4 to 6-week-old birds contained CP 18.5%, Ca 1.04%, and AP 0.241%, while diets for 7 to 16-week-old birds maintained CP 15.5%, Ca 1.01%, and AP 0.220% (Table 1). The temperature inside the experimental house was initially set at 27°C. It was then gradually decreased by 2°C each week until reaching a final temperature of 21°C. The relative humidity was maintained at a constant level of 50±10% during the whole experiment period. All experimental diets were provided in mash form throughout the trial period. Birds were given free access to feed via rectangle feeders. Fresh drinking water was provided via a nipple drinker system.

### Productive performances

To evaluate productive performances, weight gain (WG), feed intake (FI), and feed conversion ratio (FCR) were measured. All birds from the four dietary groups were individually weighed to determine WG every four weeks. The FI was calculated by subtracting the leftover feed from the total feed provided to each treatment group. To determine the FCR, total FI per pen was divided by the total WG per pen. Ages at egg laying were recorded for each pen. After 16 weeks old, birds were fed a commercial diet (2,800 kcal AME_n_/kg, 14.0% CP, 1.20% Ca, and 0.45% AP in calculated composition) to assess laying performance. The laying age at first egg was recorded. Eggs were laid for three consecutive days with an egg production of 30% or more, which was recorded as age at 30% egg production.

### Liver characteristics and abdominal fat

At 8 and 16 weeks of age, six birds from each dietary group were randomly selected. BW was measured before sacrificing them via exsanguination. After livers were collected and weighed, samples were analyzed to confirm fat content and distribution using a Digital Diagnostic X-Ray unit (Inalyzer, #XRB80N100X4391; Medikors Inc., Seongnam, Korea). The liver fat content was quantified using Dual Energy X-ray Absorptiometry (DXA) with two energy levels, 55 kV and 80 kV. This technique allows for the differentiation of fat and lean tissues by analyzing the differences in X-ray absorption between the two energy beams. The software calculated the liver fat content by comparing the attenuation differences with pre-established calibration standards, ensuring accurate quantification of fat content and distribution within the liver tissue. Liver color parameters (lightness, redness, and yellowness) were measured using a colorimeter (JP/CR-400; Minolta, Osaka, Japan). Abdominal fats of birds were collected meticulously and weighed at 16 weeks of age. The abdominal fat was calculated as a percentage (%) of the BW.

### Blood sampling and measurements

At 16 weeks of age, blood samples were taken from wing veins of six birds selected from each group. The collected blood was centrifuged at 2,000×g and 4°C for 20 minutes, and the supernatant (serum) was separated. Biochemical compositions (total cholesterol [TC], triglyceride [TG], glucose [GLU], total protein [TP], aspartate aminotransferase [AST], alanine aminotransferase [ALT]) of the serum were analyzed using a hematology analyzer (AU480 Chemistry Analyzer; Beckman Coulter Inc., Brea, CA, USA).

### Statistical analysis

All statistical analyses were conducted with one-way analysis of variance (ANOVA) procedure in SAS software version 9.4 (SAS Institute Inc., Cary, NC, USA). Duncan’s multiple range test was employed to assess significant (p<0.05) differences among dietary groups. Results are presented as mean values with pooled standard errors.

## RESULTS

### Productive performances

Productive performances of WMD1 breeder pullets fed different dietary AME_n_ levels are summarized in Tables 2. Overall, both BW and WG from 4 to 16 weeks of age were higher (p<0.05) in groups fed dietary SME-100, SME, and SME+100 than in the group fed dietary SME-200. There were no dietary group differences in FI during this experimental period. Pullets fed diets containing SME+100 and SME demonstrated significantly (p<0.05) improved FCR compared to those fed a diet containing SME-200. There was no significant difference in age at first egg laying among groups. However, the age at 30% egg production occurred was significantly (p<0.05) earlier in SME-100, SME, and SME+100 groups than in the SME-200 group.

### Fatty liver

**Figure 2:**
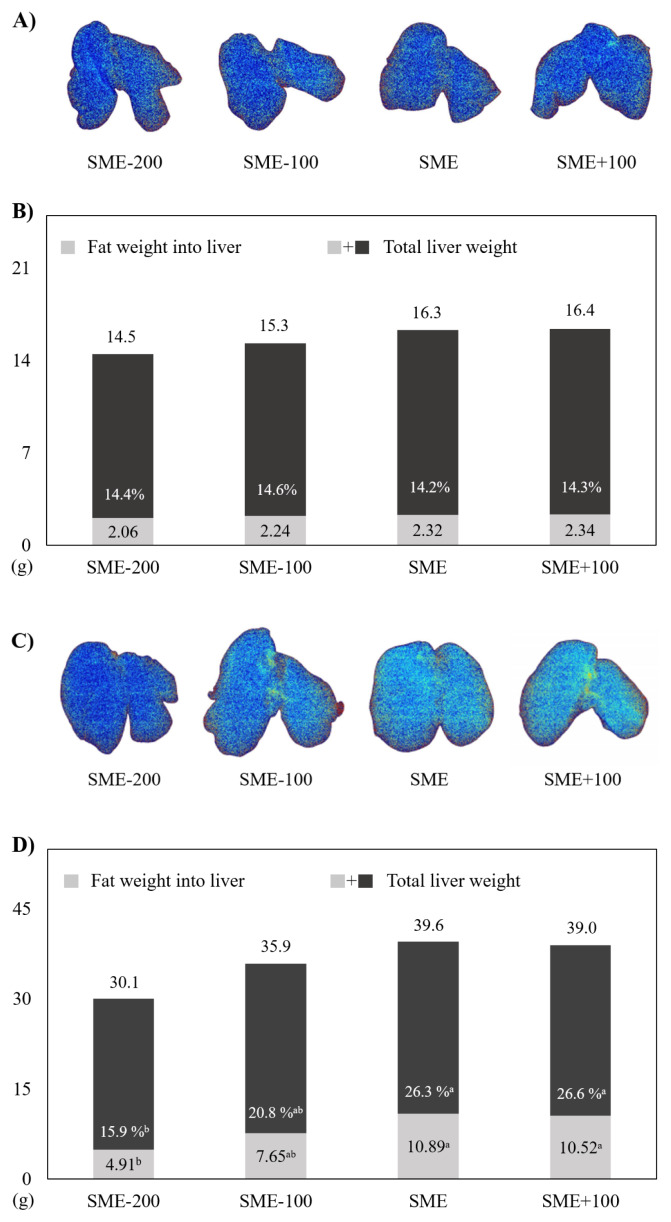
Effects of various dietary energy levels on fat deposition into livers of Woorimatdag1 breeder pullets. (A), (C) Images of the liver at 8 and 16 weeks of age analyzed using a Digital Diagnostic X-Ray unit (Inalyzer, #XRB80N100X4391; Medikors Inc., Seongnam, Korea). The redder the liver, the higher the fat content in the tissue. (B), (D) Fat contents in livers at 8 and 16 weeks of age. The gray portion and the entire bar graph indicate fat weight and total liver weight, respectively. Standard apparent metabolizable energy (SME) is 2,800 kcal/kg under the National Institute of Animal Science [18]. ^a,b^ Means sharing different superscripts differ significantly (p<0.05).

Figure 2 shows fatty liver status of pullets fed various dietary AME_n_ levels. Liver images of pullets at 8 weeks old similarly appeared in a blue color among groups (Figure 2A). Fat contents in livers showed similar results (Figure 2B). However, as the dietary AME_n_ level increases at 16 weeks of age, images of livers appear to have red color or red spots, indicating a higher fat distribution (Figure 2C). Indeed, our results indicated that birds fed SME and SME+100 diets had significantly (p<0.05) higher fat contents in livers compared to those fed the SME-200 diet at 16 weeks of age (Figure 2D).

### Liver color and abdominal fat

**Figure 3:**
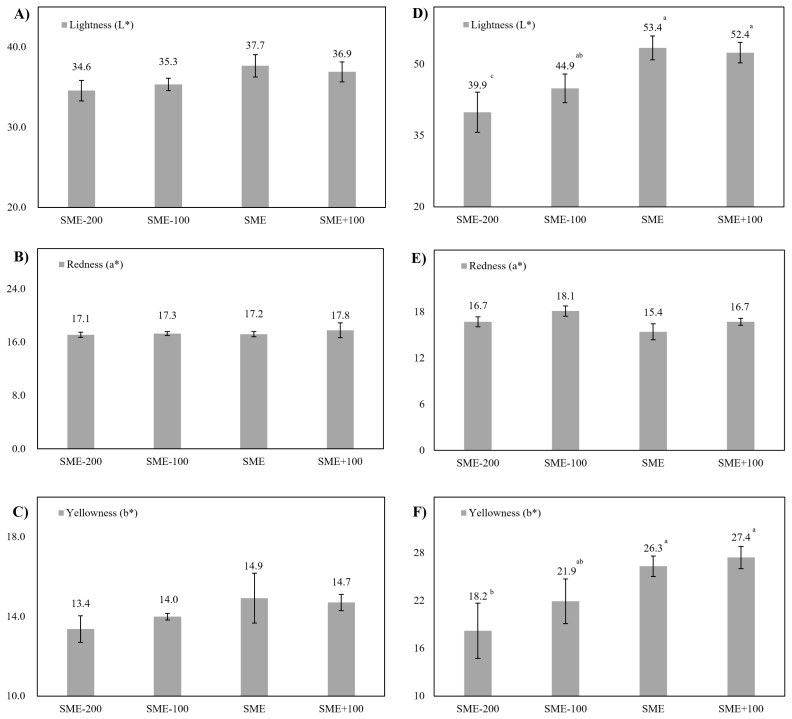
Effects of various dietary energy levels on liver color of Woorimatdag1 breeder pullets. Liver colors (lightness, redness, and yellowness) of Woorimatdag1 breeder pullets at 8 (A–C) and 16 (D–F) weeks of age are shown. Standard apparent metabolizable energy (SME) is 2,800 kcal/kg under the National Institute of Animal Science [18]. ^a–c^ Means sharing different superscripts differ significantly (p<0.05). Data are presented as mean±standard error.

**Figure 4:**
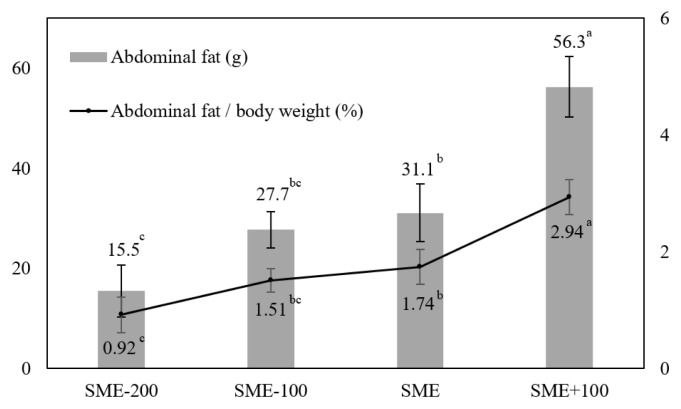
Effects of various dietary energy levels on abdominal fat deposition of Woorimatdag1 breeder pullets. Standard apparent metabolizable energy (SME) is 2,800 kcal/kg under the National Institute of Animal Science [18]. ^a–c^ Means sharing different superscripts differ significantly (p<0.05). Data are presented as mean±standard error.

There were no significant differences in liver color (lightness, redness, yellowness) among dietary groups at 8 weeks of age (Figures 3A–3C). However, as depicted in Figures 3D–3F (16 weeks of age), both the SME and SME+100 groups showed a significant (p<0.05) increase in liver lightness compared to the SME-200 group. Additionally, SME and SME+100 diets significantly (p<0.05) increased liver yellowness in pullets compared to the SME-100 diet. Furthermore, the SME+100 group had higher (p<0.05) abdominal fat (g) and abdominal fat per BW (%) than SME, SME-100, and SME-200 groups (Figure 4).

### Blood biochemical parameters

Table 3 shows results of blood biochemical parameters of birds according to dietary AME_n_ level. Results revealed no significant difference in blood composition (TC, TG, GLU, TP, AST, and ALT) among groups fed various dietary AME_n_ levels.

## DISCUSSION

Dietary AME_n_ concentrations in broilers and laying hens used in commercial poultry farming have been extensively studied [17,18,24]. Energy requirements for poultry vary depending on breeds, ages, dietary nutrient levels, and so on due to differences in nutrient digestion and absorption processes [25–27]. There are genetic differences among native Korean chicken breeds in terms of BW, meat quality characteristics, and appearance [8,9], and these genetic differences suggest the need for breed-specific feeding strategies. Recent studies have emphasized the necessity to research dietary AME_n_ levels of Native chicken breeds [28,29]. Our study aimed to determine the optimal energy of WMD1 breeder pullets.

### Productive performances

Our results indicated groups fed 2,700 to 2,900 kcal/kg had higher BWs than the group fed 2,600 kcal/kg. These findings were partially similar with those reported by Choo et al [20]. They found that a diet with 2,650 to 2,800 kcal AME_n_/kg had no effect on BW of WMD breeder hens aged 20 to 32 weeks. While the energy content in poultry diet typically correlates linearly with BW, it has been observed that levels surpassing birds’ requirements do not significantly influence BW or their digestibility [30,31]. Both BW and dietary energy management for maturation of breeders and laying pullets have emerged as key determinants influencing egg production during the laying phase [22,23,32]. Based on our results of 30% egg production, birds fed SME-100, SME, and SME+100 diets matured approximately 7 days earlier than those fed the SME-200 diet. Consequently, our research suggests that the SME-100 diet with a minimum AME_n_ level may prove to be efficient in terms of weight management of WMD1 breeder pullets. However, since our study focused on the pullet stage, further research is needed to investigate these effects during the laying period of WMD1 breeder hens, with particular attention to egg production and their economic implications.

### Fat deposition

Fatty liver disease (FLD) primarily affects poultry, characterized by excessive fat accumulation syndrome within the liver. This syndrome can lead to reduced egg production and egg quality in hens [33,34]. In severe cases, it may result in sudden death of hens due to liver rupture [35,36]. Since breeder and laying hens are typically raised until 70 to 80 weeks old through various rearing phases on poultry farms, it is crucial to regulating nutritional management of their diet from the grower phase onwards to mitigate the potential onset of FLD at laying phase [35,37,38]. In this study, fat content and intracellular fat distribution in the liver of WMD1 breeder pullets showed significant increase in SME and SME+100 groups, which might be related to the dietary energy level reported in previous studies [35,38,39]. Furthermore, the FLD of laying hens is characterized by initially presenting a yellowish color in the pale liver. However, in severe cases, it is marked by enlarged blood vessels and significant hemorrhaging [37,40]. In our results from 16-week-old, we observed higher liver color of lightness and yellowness in groups fed with SME and SME+100 diets compared to those fed with SME-200 and SME-100 diets. These results (fat content and color) may indicate a higher risk of FLD during the laying period of WMD1 breeder hens [35]. Accordingly, we recommend the SME-100 (2,700 kcal/kg) level during the grower phase (4 to 16 weeks) to mitigate the potential onset of FLD during the laying phase (17 weeks~). In our results, abdominal fat (g or %) was the highest in the SME+100 group formulated with the highest AME_n_ level, which might have partly contributed to the difference in BW. Similarly, Sunder et al [30] have reported that the abdominal fat (%) of broiler breeder pullets (5 to 20 weeks of age) is increased significantly when the dietary energy concentration is increased from 2,080 to 2,860 kcal/kg. Furthermore, recent research studies suggested that a high-energy diet can upregulate the mRNA expression of the apolipoprotein B (APOB), fatty acid-binding protein 3 (FABP3), fatty acid synthase (FASN), and lipoprotein lipase (LPL) genes, which can increase the deposition of intramuscular fat or abdominal fat [41,42]. The major goal of the poultry feed industry is to reduce carcass fatness, mainly abdominal fat, as it is seen as wasted dietary energy with limited economic value [43]. Especially, excessive accumulation of abdominal fat of broiler breeder pullets may adversely affect egg production during the laying phase due to difficulties for recommended BW targets [44]. Our results suggest that dietary energy levels below SME (2,800 kcal/kg) could alleviate abdominal fat accumulation of birds.

### Blood biochemical parameters

We confirmed that increasing energy level in the diet from 2,600 to 2,900 kcal/kg did not affect blood parameters of pullets. Results of blood parameters are supported by previous studies [42,45] showing no significant difference in blood concentration of laying hens (Hy-Line Brown) or Yangzhou geese according to an increase of dietary AME_n_ level. However, it has been reported that an increase from 2,700 to 2,900 kcal/kg AME_n_ in a diet for Japanese quails can increase TG and cholesterol concentrations [46]. Blood parameters serve as indicators of overall health status, reflecting physiological, pathological, and nutritional dynamics within the organism [47]. However, concentrations may fluctuate due to various factors including diet, environmental conditions, hormonal influences, gender, and poultry genetics [39,48]. Since no studies have reported blood parameters of KNCs fed different dietary energy levels, further research is required to evaluate blood parameters across diverse KNC breeds.

## CONCLUSION

Our findings showed that AMEn levels between 2,600 and 2,900 kcal/kg significantly impacted BW, FCR, sexual maturity, and fat deposition in WMD1 breeder pullets. The 2,700 kcal/kg (SME-100) diet achieved balanced growth, earlier maturity, and reduced fat deposition, while higher AMEn levels did not improve productive performance proportionally. Thus, the SME-100 diet offers the best balance between feed cost, productive performance, and fat reduction. Future studies could confirm these results across other developmental stages and other KNC breeds for broader application.

## Figures and Tables

**Table 1:** Experimental diets of Woorimatdag1 breeder pullets

Items (%)	4–6 weeks of age				7–16 weeks of age			
	
	SME^1)^-200	SME-100	SME	SME+100	SME-200	SME-100	SME	SME+100
Corn	48.3	52.0	55.7	59.4	52.3	56.0	59.7	63.4
Soybean meal	27.3	28.4	29.5	30.6	18.1	19.3	20.4	21.6
Wheat bran	19.0	13.7	8.40	3.10	24.2	18.8	13.5	8.10
Soybean oil	2.00	2.50	3.00	3.50	2.00	2.50	3.00	3.50
Limestone	2.00	2.00	2.00	2.00	2.00	2.00	2.00	2.00
DCP	0.600	0.600	0.600	0.600	0.600	0.600	0.600	0.600
Salt	0.250	0.250	0.250	0.250	0.250	0.250	0.250	0.250
Phytase	0.100	0.100	0.100	0.100	0.100	0.100	0.100	0.100
Vit-Min premix^2)^	0.200	0.200	0.200	0.200	0.200	0.200	0.200	0.200
L-lysine	0.200	0.200	0.200	0.200	0.200	0.200	0.200	0.200
DL-methionine	0.200	0.200	0.200	0.200	0.200	0.200	0.200	0.200
Total	100							
Calculated chemical composition								
GE (kcal/kg)	4,080	4,110	4,140	4,170	4,074	4,104	4,134	4,164
AME_n_ (kcal/kg)	2,602	2,702	2,801	2,900	2,601	2,701	2,800	2,900
CP (%)	18.5				15.5			
Ca (%)	1.04				1.01			
TP (%)	0.562	0.531	0.500	0.470	0.558	0.527	0.497	0.466
AP (%)	0.241				0.220			
Lysine (%)	1.06	1.07	1.08	1.09	0.867	0.878	0.889	0.889
Methionine (%)	0.481	0.480	0.478	0.476	0.444	0.443	0.441	0.439
Tryptophan (%)	0.675	0.681	0.687	0.694	0.554	0.561	0.568	0.574
Analyzed chemical composition								
GE (kcal/kg)	4,083	4,119	4,124	4,200	4,091	4,115	4,145	4,163
CP (%)	18.4	18.4	18.8	18.4	15.5	15.2	15.3	15.6
Ca (%)	1.04	1.10	1.06	1.08	1.00	0.99	1.03	1.02

SME, standard apparent metabolizable energy; DCP, dicalcium phosphate; GE, gross energy; AME_n_, apparent metabolizable energy; CP, crude protein; TP, total phosphorus; AP, available phosphorus.

1) SME is 2,800 kcal AME_n_/kg under the National Institute of Animal Science [18].

2) Contains per kg, Vit A, 12,000 IU; Vit D3, 5,000 IU; Vit K3, 3 mg; Vit B1, 2 mg; Vit B2, 6 mg; Vit B6, 4 mg; Vit B12, 25 mg; biotin, 0.2 mg; folic acid, 0.2 mg; niacin, 70 mg; pantothenic acid, 20 mg; Cu, 20 mg; Co, 0.5 mg; Fe, 50 mg; I, 1,300 mg; Mn, 120 mg; Se, 0.3 mg; Zn, 100 mg..

**Table 2:** Effects of various dietary energy levels on productive performances of Woorimatdag1 breeder pullets

Dietary groups	SME^1)^-200	SME-100	SME	SME+100	SEM	p-value
4–8 weeks of age						
BW (g)	655^b^	676^ab^	662^b^	694^a^	5.11	0.020
WG (g)	438^b^	460^ab^	446^b^	480^a^	5.25	0.013
FI (g)	1,297	1,307	1,274	1,318	14.0	0.756
FCR	2.96	2.84	2.86	2.75	0.036	0.200
9–12 weeks of age						
BW (g)	1,159^b^	1,233^a^	1,218^a^	1,265^a^	12.5	0.011
WG (g)	783^b^	859^a^	851^a^	876^a^	12.1	0.020
FI (g)	2,021	2,118	2,053	2,087	17.3	0.223
FCR	2.58a	2.47^ab^	2.42^b^	2.38^b^	0.026	0.024
13–16 weeks of age						
BW (g)	1,667^b^	1,787^a^	1,775^a^	1,819^a^	17.5	0.004
WG (g)	752	801	818	801	9.76	0.079
FI (g)	2,502	2,618	2,495	2,502	23.1	0.177
FCR	3.33	3.27	3.06	3.13	0.046	0.116
Total (4–16 weeks of age)						
BW (g)	1,667^b^	1,787^a^	1,775^a^	1,819^a^	17.5	0.004
WG (g)	1,450^b^	1,571^a^	1,559^a^	1,605^a^	17.6	0.003
FI (g)	5,820	6,044	5,823	5,907	40.1	0.159
FCR	4.01^a^	3.85^ab^	3.74^b^	3.68^b^	0.040	0.005
Age of egg production (d)^2)^						
First egg	116.8	117.4	118.2	115.0	0.612	0.312
30% egg production	134.0^a^	127.0^b^	127.2^b^	128.8^b^	0.954	0.017

SME, standard apparent metabolizable energy; SEM, standard error of the mean; BW, body weight; WG, weight gain; FI, feed intake; FCR, feed conversion ratio.

1) SME is 2,800 kcal/kg under the National Institute of Animal Science [18].

2) First egg, initial egg laying age; 30% egg production, 3 consecutive days with an egg production (%) of 30% or higher

a,b Means in a row sharing different superscripts differ significantly (p<0.05).

**Table 3:** Effects of various dietary energy levels on biochemical parameters of Woorimatdag1 breeder pullets

Dietary groups	SME^1)^-200	SME-100	SME	SME+100	SEM	P value
TC (mg/dL)	103	119	123	113	5.72	0.672
TG (mg/dL)	269	275	262	271	35.5	0.998
GLU (mg/dL)	201	209	180	187	5.34	0.207
TP (g/dL)	4.20	4.33	4.05	4.20	0.116	0.893
AST (U/L)	207	212	222	217	6.46	0.891
ALT (U/L)	1.47	1.46	1.40	1.49	0.090	0.990

SME, standard apparent metabolizable energy; SEM, standard error of the mean; TC, total cholesterol; TG, triglyceride; GLU, glucose; TP, total protein; AST, aspartate aminotransferase; ALT, alanine aminotransferase.

1) SME is 2,800 kcal/kg under the National Institute of Animal Science [18].
